# Integrated Transcriptomics and Widely Targeted Metabolomics Analyses Provide Insights Into Flavonoid Biosynthesis in the Rhizomes of Golden Buckwheat (*Fagopyrum cymosum*)

**DOI:** 10.3389/fpls.2022.803472

**Published:** 2022-06-17

**Authors:** Juan Huang, Luyuan Wang, Bin Tang, Rongrong Ren, Taoxiong Shi, Liwei Zhu, Jiao Deng, Chenggang Liang, Yan Wang, Qingfu Chen

**Affiliations:** ^1^Research Center of Buckwheat Industry Technology, Guizhou Normal University, Guiyang, China; ^2^Tunliu District Vocational Senior Middle School, Changzhi, China

**Keywords:** *Fagopyrum cymosum* rhizome, transcriptomics, flavonoid biosynthesis, MYB transcription factor, widely targeted metabolomics

## Abstract

Golden buckwheat (*Fagopyrum cymosum*) is used in Traditional Chinese Medicine. It has received attention because of the high value of its various medicinal and nutritional metabolites, especially flavonoids (catechin and epicatechin). However, the metabolites and their encoding genes in golden buckwheat have not yet been identified in the global landscape. This study performed transcriptomics and widely targeted metabolomics analyses for the first time on rhizomes of golden buckwheat. As a result, 10,191 differentially expressed genes (DEGs) and 297 differentially regulated metabolites (DRMs) were identified, among which the flavonoid biosynthesis pathway was enriched in both transcriptome and metabolome. The integration analyses of the transcriptome and the metabolome revealed a network related to catechin, in which four metabolites and 14 genes interacted with each other. Subsequently, an SG5 R2R3-MYB transcription factor, named *FcMYB1*, was identified as a transcriptional activator in catechin biosynthesis, as it was positively correlated to eight flavonoid biosynthesis genes in their expression patterns and was directly bound to the promoters of *FcLAR2* and *FcF3'H1* by yeast one hybrid analysis. Finally, a flavonoid biosynthesis pathway was proposed in the rhizomes of golden buckwheat, including 13 metabolites, 11 genes encoding 9 enzymes, and 1 MYB transcription factor. The expression of 12 DEGs were validated by qRT-PCR, resulting in a good agreement with the Pearson R ranging from 0.83 to 1. The study provided a comprehensive flavonoid biosynthesis and regulatory network of golden buckwheat.

## Introduction

Common buckwheat (*Fagopyrum esculentum* Moench), Tartary buckwheat (*Fagopyrum tataricum* (L.) Gaertn), and golden buckwheat (*Fagopyrum cymosum*, also called *Fagopyrum dibotrys*) are three major species of the genus *Fagopyrum* Mill in the *Polygonum* family (Ohnishi and Matsuoka, 1996; Chen et al., 2018). Among them, common buckwheat and Tartary buckwheat are utilized as food or feed and mostly found as diploid, whereas golden buckwheat is utilized as medicine or tea and found as diploid or tetraploid (Mazza and Oomah, 2003; Gu et al., 2014; Chen et al., 2018). Golden buckwheat is one of the National Key Protected Wild Plants (Class II) in China, and its rhizome is a traditional Chinese medicine included in the Chinese Pharmacopoeia (2020) with high medicinal value (Chiang et al., 2000). Golden buckwheat is also known as a health food because it has high nutritional value and healthcare function. Therefore, it is utilized to make healthy products such as fermented tea and root slices (Yang et al., 2007; Gu et al., 2014). The rhizome, as the medicinal tissue of golden buckwheat, is rich in various medicinal and hygienical components such as flavonoids (catechin and epicatechin), antioxidant phenolic compounds, γ-aminobutyric acid, and terpenoids (Shao et al., 2005; Wang et al., 2005; Zhang et al., 2016). Among these, flavonoids are some of the most functionally valuable components beneficial to health that are good for gastrointestinal dysfunction; enhance vascular toughness; reduce blood sugar, blood fat, and inflammation; promote tumor inhibition; and improve immunity (Chan, 2003; Li et al., 2021).

Proanthocyanidins are plant polyphenols formed by the condensation of flavane-3-ol monomers such as catechin and epicatechin (Li et al., 2021). They are the last components in flavonoid biosynthesis and essential bioactive components in golden buckwheat. The content of epicatechin is a medicinal indicator for evaluating the quality of the rhizomes of golden buckwheat in the Chinese Pharmacopoeia (2020) (Chiang et al., 2000). Therefore, more and more research has focused on flavonoids in golden buckwheat in recent years. Li et al. (2021) purified proanthocyanidins and analyzed their detailed structure in golden buckwheat rhizome. The contents, antioxidant activity, and antidiabetic activity in golden buckwheat rhizome were higher than those in six other *Polygonaceae* plants (Li et al., 2021). Chen et al. obtained a golden buckwheat mutant that had higher epicatechin content and elucidated the molecular mechanism underlying proanthocyanidin accumulation by transcriptomes in which unigenes in radiation-mediated flavonoid biosynthesis were identified (Jia and Li, 2008; Chen et al., 2012; Chen and Li, 2016). Besides, several genes in the flavonoid biosynthesis of golden buckwheat were cloned and analyzed, including *FdPAL, FdFLS, FdCHI, FdCHS, FdDFR, FdANS, FdLAR*, and *FdMYB* (Liu et al., 2009; Ma et al., 2009, 2012; Meng et al., 2010; Li et al., 2011; Jiang et al., 2013; Luo et al., 2013; Pu et al., 2014).

Although the flavonoid biosynthesis pathway and regulatory network in golden buckwheat are far from clear, they have been thoroughly uncovered in many other plants (Saito et al., 2013; Kelemen et al., 2015; Xu et al., 2015; Tohge et al., 2017). There are more than 6,000 identified flavonoids in plants, which have diverse biological functions, including plant defense, signaling during nodulation, and flower and fruit coloration (Falcone Ferreyra et al., 2012; Tohge et al., 2018; Zhao et al., 2020; Bosse et al., 2021). The biosynthesis pathway of flavonoids initiates from the formation of malonyl-CoA and p-coumaroyl-CoA, among which, malonyl-CoA is catalyzed by acetyl-CoA carboxylase, and p-coumaroyl-CoA is catalyzed by phenylalanine ammonialyase (PAL), cinnamate 4-hydroxylase (C4H), and 4-coumarate CoA ligase (4CL). Then malonyl-CoA and p-coumaroyl-CoA are converted into flavonoid scaffolds, including dihydroflavonols and the subsequently formed proanthocyanidins, by a complex series of reactions. Dihydroflavonols are catalyzed by chalcone synthase (CHS), chalcone isomerase, flavanone 3-hydroxylase (F3H), flavonoid 3′-hydroxylase (F3′H), flavonoid 3′,5′-hydroxylase (F3′5′H), and flavonol synthase (FLS). Proanthocyanidins are catalyzed by dihydroflavonol 4-reductase (DFR), leucoanthocyanidin reductase (LAR), and anthocyanidin reductase (Saito et al., 2013; Chen and Li, 2016; Tohge et al., 2017). Massive flavonoid biosynthesis and regulation are characterized by involving enzyme encoding genes and families of transcription factors, including R2R3-MYB, bHLH, and WD40 proteins, and their interactions (MYB-bHLH-WD40 complex) (Kelemen et al., 2015; Xu et al., 2015; Zhang et al., 2018, 2019; Li et al., 2019c; Sun et al., 2020; Yao et al., 2020; Ding et al., 2021).

Previously, we collected 211 golden buckwheat accessions from different provinces in China. They were divided into three categories based on total flavonoid content (Wang et al., 2019). The current study aimed to make up for the deficiency of flavonoid biosynthesis pathways and regulatory networks in golden buckwheat. We performed transcriptome and widely targeted metabolome analyses using four golden buckwheat accessions that differed in total flavonoid content. Massive differentially expressed genes (DEGs) and differentially regulated metabolites (DRMs) were identified. Integration analyses of the transcriptome and metabolome profiles were carried out, and a flavonoid biosynthesis pathway in the rhizomes of golden buckwheat was proposed. A yeast one-hybrid (Y1H) analysis was conducted to validate the binding of an MYB transcription factor in promoters of flavonoid biosynthesis genes. A quantitative reverse transcription PCR (qRT-PCR) analysis of 13 DEGs was performed to verify the transcriptome results. This study provided a comprehensive flavonoid biosynthesis and regulatory network of golden buckwheat.

## Materials and Methods

### Plant Growth and Sampling

Four golden buckwheat collections were used as plant materials in this study, namely, R1, R2, R3, and R4. Among these, R1 originated from Shangri-la, Yunnan province (longitude 100°05′, latitude 27°19′, and altitude 3,452 m); R2 originated from Kunming City, Yunnan province (longitude 102°82′, latitude 24°89′, and altitude 1,916 m); R3 originated from Kaiyang county, Guizhou province (longitude 106°96′, latitude 27°06′, and altitude 1,228 m); R4 was kept by our laboratory. They were planted by cutting in the experimental field in March 2017, and with normal field management during the growth periods. For transcriptome and widely targeted metabolome analyses, the golden yellow rhizomes were sampled and washed by ddH_2_O. The fibrous root was removed, the remaining rhizomes were cut into portions, and quickly subjected to liquid nitrogen for subsequent experiments. Three biological replicates were included for each golden buckwheat collection.

### Transcriptome Analyses

Total RNA was isolated using a plant RNA purification kit [TianGen Biotech (Beijing) Co., Ltd., China], followed by treatment with Dnase I for 20 min to digest genomic DNA. For each sample, 1-μg RNA was used for library construction, using NEBNextUltraTM RNA Library Prep Kit for Illumina (NEB, United States) according to the manufacturer's instruction. The library's quality was evaluated with the Agilent Bioanalyzer 2100 system. High-throughput transcriptome sequencing was performed on NOVASEQ 6000 System (Illumina Inc., United States) with a read length of 150 bp and a paired-end method.

Raw reads were filtered by in-house Perl scripts to obtain clean data. Then, the clean reads were mapped to the Tartary buckwheat genome data using Hisat2 (Kim et al., 2015; Zhang et al., 2017). Only reads with a perfect match or one mismatch were further analyzed and annotated based on the reference genome. Gene function annotation was performed by a local BLASTP against NR, Pfam, Swiss-Prot, KO, and GO database (1e-5). Afterward, gene expression was normalized to fragments per kilobase of transcript per million fragments mapped (FPKM) as the following: $FPKM=\frac{cDNA\;Fragments}{Mapped\;Fragments\;\left(Millions\right)\;*\;Transcript\;Length\;\left(kb\right)}$. DEGs were identified with the DESeq2 package in the R language (https://www.R-project.org/), with an adjusted *P*-value (padj) <0.05 and absolute value of log_2_ratio ≥ 1 as the thresholds (Love et al., 2014). Principal component analysis (PCA), Spearman's correlation coefficient, GO enrichment analyses, and hierarchical cluster were performed and visualized by the libraries in the R language, namely scatterplot3d, ggplot2, clusterProfiler, pheatmap, reshape2, and factoextra. The KEGG pathway was enriched with KOBAS 3.0 (http://kobas.cbi.pku.edu.cn/kobas3/?t=1).

### Widely Targeted Metabolome Analyses

Metabolite analysis was performed by Metware Biotechnology Co., Ltd. (Wuhan, China) as previously described (Chen et al., 2013; Zhao et al., 2019). The freeze-dried rhizome was crushed using a mixer mill (MM 400; Retsch) with a zirconia bead for 1.5 min at 30 Hz. Then, 100-mg powder for each sample was weighed and treated with 0.6 ml 70% aqueous methanol overnight at 4°C. Following centrifugation at 10, 000 g for 10 min, extracts were filtrated with a 0.22-μm pore size membrane and stored in chromatographic sample bottles.

Then, the sample extracts were analyzed using a UPLC-ESI-MS/MS system. The UPLC (Shim-pack UFLC SHIMADZU CBM30A system) analytical conditions were set as follows: (1) Column, Waters ACQUITY UPLC HSS T3 C18 (1.8 μm, 2.1 mm^*^100 mm); (2) mobile phase, solvent A was pure water with 0.04% acetic acid and solvent B was acetonitrile with 0.04% acetic acid; (3) gradient elution: sample measurements were performed with a gradient program that employed the starting conditions of 95% A and 5% B. Within 10 min, a linear gradient was programmed, and a composition of 5% A and 95% B was kept for 1 min. Subsequently, a composition of 95% A and 5% B was adjusted within 0.1 min and kept for 2.9 min; (4) injection volume, 4 μl; column oven, 40°C; flow rate, 0.35 ml/min.

The effluent was alternatively connected to a triple quadrupole-linear ion trap (QTRAP)-MS (Applied Biosystems 4500 QTRAP) for LIT and QQQ scan, equipped with an ESI Turbo Ion-Spray interface operating in positive and negative ion modes and controlled with the Analyst 1.6.3 software (AB Sciex). ESI source operation parameters were as follows: ion source, turbo spray; source temperature 550°C; ion spray voltage 5,500 V (positive ion mode)/−4,500 V (negative ion mode); ion source gas I, gas II, curtain gas were set at 50, 60, and 30 psi, respectively; the collision gas was high. Instrument tuning and mass calibration were performed with 10 and 100 μmol/L polypropylene glycol solutions in QQQ and LIT modes, respectively. QQQ scans were acquired as MRM experiments with collision gas (nitrogen) set to 5 psi. Declustering potential (DP) and collision energy (CE) for individual MRM transitions were conducted with further DP and CE optimization. A specific set of MRM transitions were monitored for each period according to metabolites eluted within this period (Chen et al., 2013; Zhou et al., 2019). Mass spectrometric data were processed with Analyst 1.6.3. Then, the quality and quantity of metabolites were processed with MultiaQuant based on a local metabolite database constructed by Metware Biotechnology. Quality control was performed by evaluating the repeatability of three technical replicates prepared from a mixed extract of all the samples. Then, the obtained data were log transformed (log_2_) and subjected to the MetaboAnalystR package in the R language for Pearson correlation analysis, PCA analysis, and OPLS-DA analysis (Chong and Xia, 2018). DRMs were identified by variable importance in the projection value (VIP) ≥ 1 and absolute log_2_ratio ≥ 1. A permutation test with 200 permutations was performed to avoid overfitting.

### Integration of the Transcriptome and Metabolome Profiles

The transcriptome and metabolome data were log transformed (log_2_) for integration analyses. A Pearson correlation coefficient (PCC) analysis was performed in the R language, and significantly correlated genes and metabolites were identified by PCC > 0.8 and a *P-*value of PCC (PCCP) <0.05 (Jozefczuk et al., 2010). The network of genes and metabolites with PCC > 0.8 was visualized with the software Cytoscape (Shannon et al., 2003). A canonical correlation analysis (CCA) was performed for genes and metabolites in the correlation network using the CCA package in the R language (González et al., 2008).

### Y1H Analysis

The obtaining of promoter sequences (1,500 bp) of candidate genes and scanning of DNA-binding specificities of R2R3-MYB proteins (Kelemen et al., 2015) in the promoters were performed with in-house Python programs. Y1H was performed as previously described (Li et al., 2017). Briefly, the promoters of *FcDFR1* (FtPinG0002371500.01), *FcF3H1* (FtPinG0006662600.01), *FcF3*′*H1* (FtPinG0002353900.01), *FcLAR2* (FtPinG0002428800.01), and *FcCHS2* (FtPinG0002106500.01) were cloned and linked into pHIS2 with the *EcoR*I and *Mlu*I restriction sites. The coding sequence of *FcMYB1* (FtPinG0007213700.01) was cloned and linked to pGADT7 with the *EcoR*I and *BamH*I restriction sites. Yeast Y187 was co-transformed with pGADT7-MYB and five constructs containing corresponding promoters. The co-transformed yeast was selected in an SD/–Leu/–Trp medium to test whether the co-transformations were successful. Then, an SD/–His/–Leu/–Trp medium was used to test the expression of the HIS3 reporter; 90, 110, and 150 mM 3-AT were used to inhibit the auto-activation of pHIS2 constructs with five promoters. The primer sequences described above are listed in Supplementary Table 1.

### qRT-PCR Analysis

The transcriptome results were verified by qRT-PCR. A total of 13 DEGs related to flavonoid biosynthesis were selected, namely, *FcCHS1* (FtPinG0000551600.01), *FcCHS2, FcCHS3* (FtPinG0008806400.01), *FcCHI1* (FtPinG0003061400.01), *FcF3H1, FcF3*′*H1, FcF3*′*5*′*H1* (FtPinG0006940000.01), *FcF3*′*5*′*H2* (FtPinG0006132400.01), *FcDFR1, FcFLS1* (FtPinG0006907000.01), *FcLAR1* (FtPinG0000053800.01), *FcLAR2*, and *FcMYB1*. *Actin* was used as the inner reference gene. Primer3Plus (http://www.primer3plus.com/cgi-bin/dev/primer3plus.cgi) was used to pick gene-specific primers (Supplementary Table 1). qRT-PCR was performed on an ABI 7500 Fast Real-Time PCR system (Applied Biosystems, United States) using an SYBR^®^ Premix Ex Taq™ II kit (Takara Biomedical Technology (Beijing) Co., Ltd., China). The procedure was performed in accordance with the manufacturers′ instructions, with three technical replicates. qRT-PCR results were calculated using the 2^−Δ*ΔCt*^ method.

## Results

### Transcriptome Analysis of Golden Buckwheat Rhizomes

High-throughput RNA-Seq was performed on 12 RNA samples from the rhizomes of four golden buckwheat collections (R1, R2, R3, and R4), with three biological replicates for each collection. As a result, we obtained 41,034,456–52,182,636 clean reads and 6,111,106,094–7,791,174,252 clean bases for each library. GC content ranged from 46.17–49.63%. Among these, 63.6–72.36% of clean reads were uniquely mapped to the predicted coding sequences of the genome data of Tartary buckwheat. Meanwhile, 34.3–36.98% of clean reads were mapped to the “+” strand of the coding sequences, whereas 34.04–37.01% were mapped to the “–” strand of the coding sequences (Supplementary Table 2). After mapping, 28,492 genes were identified in all the 12 libraries. Besides, new genes that were not included in the reference genome data were also identified, resulting in 1,665 new genes.

In addition, all of the 12 samples were clustered, and the repeatability of biological replicates was evaluated. As shown in Supplementary Figure 1, samples of the same biological replicates were clustered closer, with correlations ranging from 0.8503 to 0.9546, whereas samples out of the biological replicates were not closely related, with correlations ranging from 0.6777 to 0.8799, indicating our data had high reliability and reproducibility.

**Figure 1:**
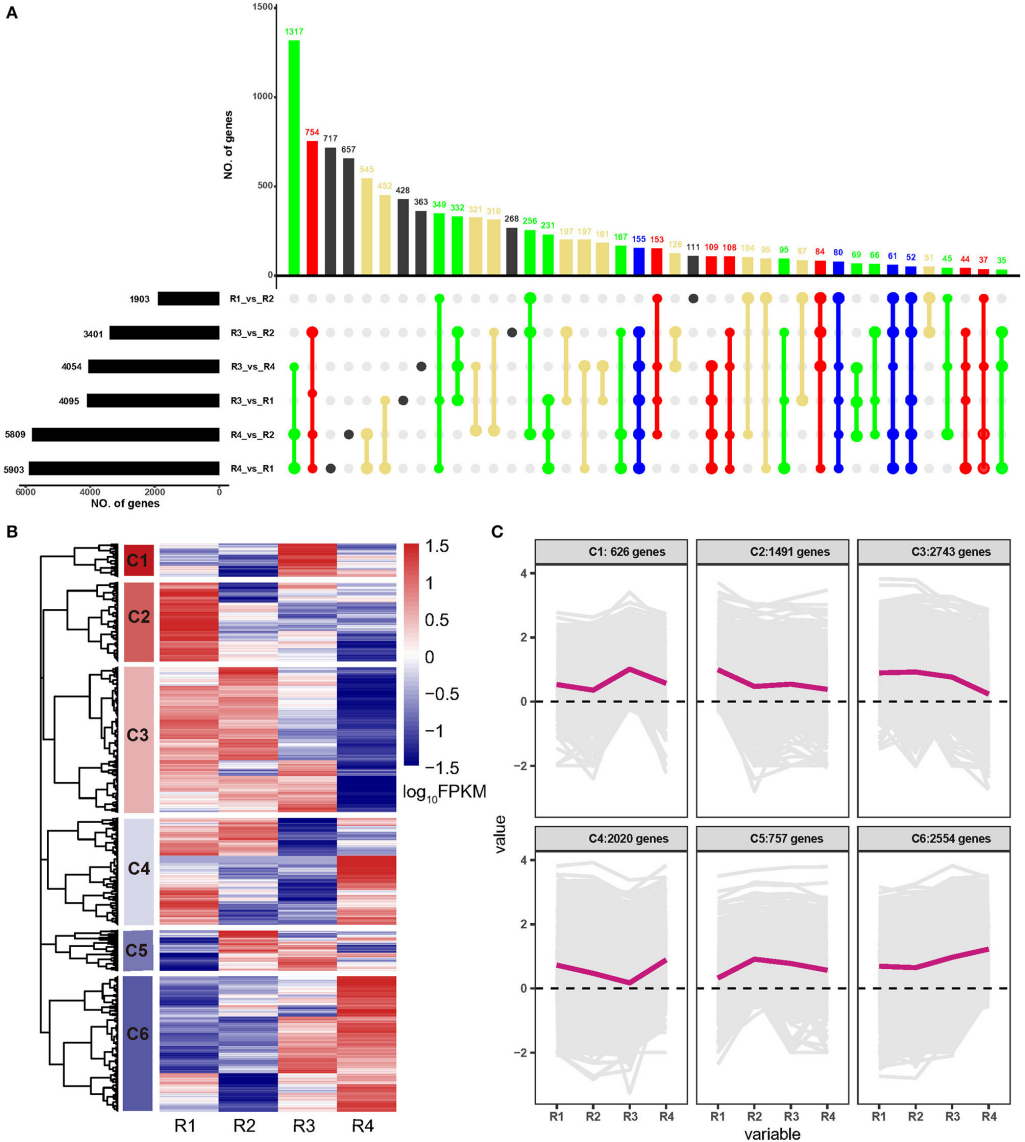
Analyses of differentially expressed genes (DEGs) of golden buckwheat rhizomes. **(A)** The upset plot of the DEGs in two out of six comparisons. Black bars on the lower left represent the numbers of DEGs in comparisons R1, R2, R3, and R4. The colored bars above represent the number of common genes. Blue dots represent genes that are common in at least five of the six comparisons. Red dots represent genes that are common in at least four of the six comparisons. Green dots represent genes that are common in at least three of the six comparisons. Yellow dots represent genes that are common in at least two of the six comparisons. Black dots represent genes that are differentially regulated in only one of the six comparisons. **(B)** Functional category of DEGs by hierarchical cluster. **(C)** Expression patterns of the six clusters correspond to the hierarchical cluster result. Six main clusters are presented as C1–C6. Gene expression values are normalized to log_10_(FPKM).

A total of 10,191 DEGs were identified in all the samples, among which 1,903, 3,401, 4,054, 4,095, 5,809, and 5,903 were identified in R4_vs._R1, R4_vs._R2, R3_vs._R1, R3_vs._R4, R3_vs._R2, and R1_vs._R2, respectively (Supplementary Table 3 and Figure 1A). Of these, 155 DEGs were common in five comparisons except for R1_vs._R2; 80 DEGs were common in five comparisons except for R3_vs._R2; 61 DEGs were common in five comparisons except for R3_vs._R1, and 52 DEGs were common in five comparisons except for R3_vs_R4 (Figure 1A). Besides, a hierarchical cluster analysis based on gene expression pattern was performed and six clusters, namely, C1 to C6, were obtained (Figures 1B,C). Among these, C1 included 626 genes that were upregulated in R3; C2 included 1,492 genes that were upregulated in R1; C3 included 2,743 genes that showed higher expression levels in R1, R2, and R3 but lower expression levels in R4; C4 included 2,020 genes that showed higher expression level in R1, R2, and R4 but lower expression level in R3; C5 included 757 genes that showed higher expression level in R2, R3, and R4 but lower expression level in R1; and C6 included 2,554 genes that showed higher expression level in R3 and R4 but lower expression level in R1 and R2.

A GO analysis was performed, and DEGs in the six comparisons were classified into three component categories. The top 30 GO terms are presented in Supplementary Figure 2. Interestingly, some biological processes related to the pathway of flavonoid biosynthesis and metabolism, such as “flavonoid biosynthetic process,” “flavonol biosynthetic process,” “flavone biosynthetic process,” and “flavonoid glucuronidation,” were enriched in at least two comparisons. At the same time, we analyzed the metabolic pathways based on KEGG annotation. Among the top 20 most enriched pathways in the six comparisons (Supplementary Figure 3), “flavonoid biosynthesis,” “flavone and flavonol biosynthesis,” and “phenylpropanoid biosynthesis” also took place. These implied that flavonoids might show some difference among four golden buckwheat rhizomes.

### Metabolomics Analyses of Golden Buckwheat Rhizomes

**Figure 2:**
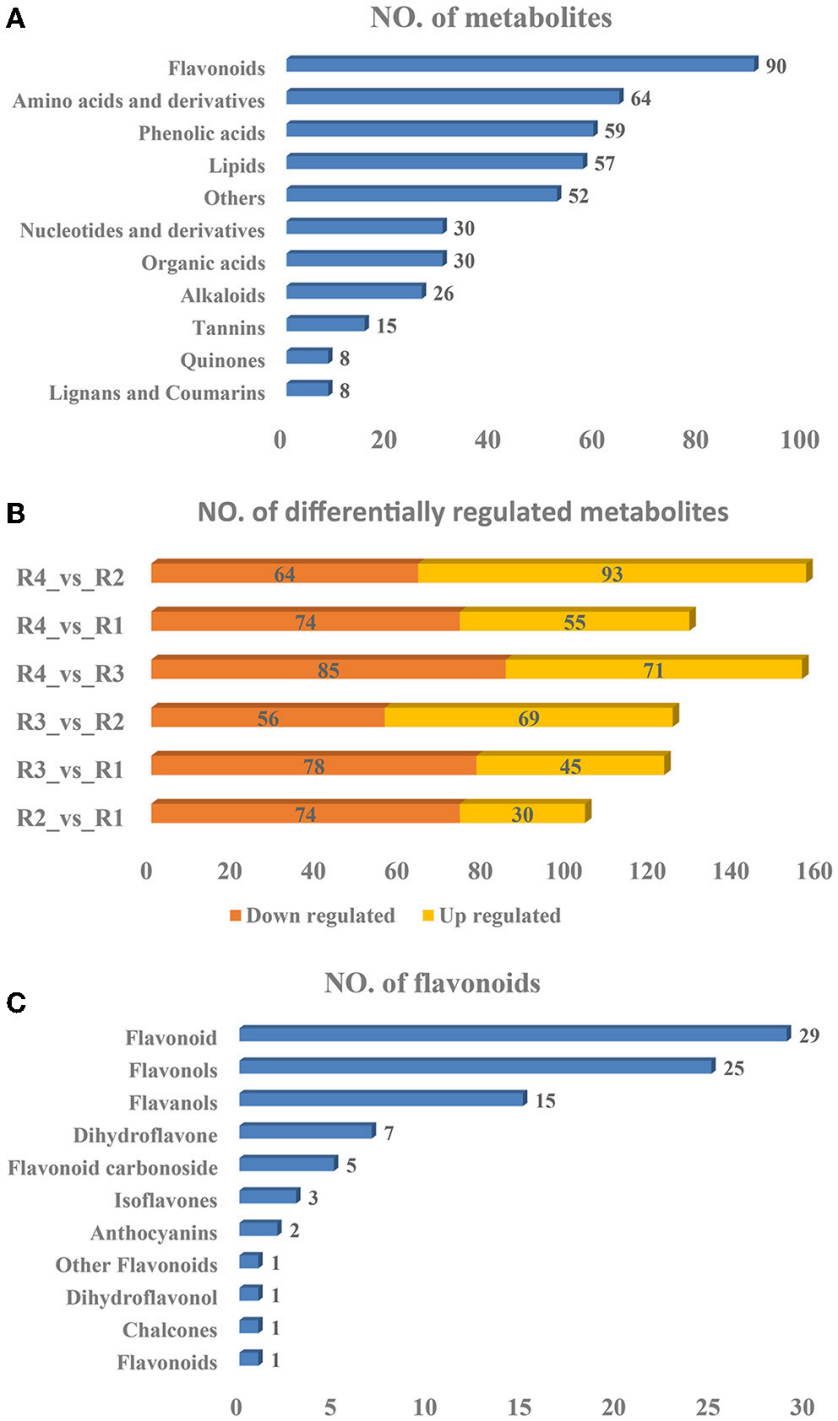
Statistics of the **(A)** identified metabolites, **(B)** differentially regulated metabolites, and **(C)** flavonoids of golden buckwheat rhizomes based on metabolomics analyses.

Widely targeted metabolomics analyses were subsequently performed to identify the metabolites existing in golden buckwheat rhizomes, resulting in 439 metabolites being identified (Supplementary Table 4). These included 90 flavonoids, 64 amino acids and derivatives, 59 phenolic acids, 57 lipids, 52 others, 30 organic acids, 30 nucleotides and derivatives, 26 alkaloids, 15 tannins, 8 lignans and coumarins, and 8 quinones (Figure 2A). The PCA analysis showed that samples of the same biological replicates were less variable than those of the biological replicates, suggesting that the metabolomics data had high reliability and reproducibility (Supplementary Figure 4).

A total of 297 DRMs were identified in all the samples, among which 93, 55, 71, 69, 45, and 30 were upregulated in the comparisons R4_vs._R2, R4_vs._R1, R4_vs._R3, R3_vs._R2, R3_vs._R1, and R2_vs_R1, respectively, whereas 64, 74, 85, 56, 78, and 74 were downregulated in the comparisons R4_vs._R2, R4_vs._R1, R4_vs._R3, R3_vs._R2, R3_vs._R1, and R2_vs._R1, respectively (Figure 2B and Supplementary Table 5). All the DRMs were clustered into nine subclasses based on K means cluster analysis (Supplementary Figure 5). Metabolites in different subclasses showed different regulation patterns: subclass 1 included 58 metabolites that were upregulated in R4, subclass 2 included 37 metabolites that were upregulated in R1, subclass 3 included 23 metabolites that were upregulated in R2 and R3, subclass 4 included 26 metabolites that were downregulated in R3 but were upregulated in R4, subclass 5 included 21 metabolites that were upregulated in R2, R3, and R4, subclass 6 included 32 metabolites that were upregulated in R3, subclass 7 included 26 metabolites that were upregulated in R3 but were downregulated in R4, subclass 8 included 52 metabolites that were upregulated in R2. Subclass 8 also included 52 metabolites that were downregulated in R2. In addition, KEGG metabolic pathways based on the DRMs were analyzed. Similar to the transcriptomics result, “flavonoid biosynthesis,” “flavone and flavonol biosynthesis,” and “phenylpropanoid biosynthesis” were also listed in the top 20 significantly enriched pathways in all of the six comparisons (Supplementary Figure 6). These indicated that flavonoids might be the most essential compounds in golden buckwheat rhizomes.

It is well-known that some kind of flavonoids, such as epicatechin, is the most essential index for evaluating whether golden buckwheat rhizomes are up to the standard of Chinese medicinal material (Chiang et al., 2000). In all kinds of identified metabolites, flavonoids accounted for the most abundant one, so we subsequently analyzed the type of flavonoids. Among the 90 flavonoids, 29 flavonoids, 25 flavonols, 15 flavanols, 7 dihydroflavones, 5 flavonoid carbonosides, 3 isoflavones, 2 anthocyanins, 1 flavonoid, 1 chalcone, 1 dihydroflavonol, and 1 other flavonoid were included (Figure 2C).

The top 20 abundant flavonoids were compared, among which 18 were identified as DRMs. These included 6 flavonols (6-hydroxykaempferol-7-o-glucoside, syringetin 3-o-hexoside, quercetin 3-o-rhanosylgalactoside, quercetin-3-o-glucoside-7-o-rhamnoside, kaempferol 3,7-dirhamnoside(kaempferitrin), and bioquercetin), 5 flavonoids (nepetin, tricin 7-o-hexoside, luteolin 8-c-hexosyl-o-hexoside, chrysoeriol, and quercetin-p-coumaroyl hexose), 4 flavanols (catechin, gallic acid, (-)-epicatechin gallate, and (-)-catechin gallate), 2 dihydroflavones (hesperidin and eriodictyol c-hexoside), and 1 isoflavone (pratensein) (Table 1).

**Table 1:** Top 20 Flavonoids identified in golden buckwheat rhizomes.

**Index**	**Retention time (min)**	**Molecular weight (Da)**	**Formula**	**Ionization model**	**Compounds**	**Class II**	**Log_2_(R2/R1)**	**log_2_(R3/R1)**	**log_2_(R4/R1)**	**log_2_(R3/R2)**	**log_2_(R4/R2)**	**log_2_(R4/R3)**
mws0054	3.5	290.1	C_15_H_14_O_6_	[M–H]^−^	Catechin	Flavanols	−0.1	−1.0	−0.9	−0.9	−0.8	0.1
mws0024	2.2	170.0	C_7_H_6_O_5_	[M–H]^−^	Gallic acid	Flavanols	−1.2	−3.6	−0.7	−2.4	0.5	2.9
mws0036	4.5	610.2	C_28_H_34_O_15_	[M–H]^−^	Hesperidin	Dihydroflavone	1.2	1.8	1.9	0.6	0.7	0.1
pmp000003	5.7	316.0	C_16_H_12_O_7_	[M+H]^+^	Nepetin	Flavonoid	2.3	2.3	−0.5	−0.1	−2.9	−2.8
pme0460	3.8	290.1	C_15_H_14_O_6_	[M+H]^+^	L-Epicatechin	Flavanols	0.0	−0.1	0.4	0.0	0.4	0.5
pmp001309	4.2	464.1	C_21_H_20_O_12_	[M+H]^+^	6-Hydroxykaempferol-7-O-glucoside	Flavonols	1.6	2.2	1.9	0.6	0.3	−0.3
pmb0565	3.9	508.1	C_23_H_24_O_13_	[M+H]^+^	Syringetin 3-O-hexoside	Flavonols	3.7	2.0	1.3	−1.7	−2.4	−0.7
mws0183	3.0	154.0	C_7_H_6_O_4_	[M-H]^−^	Protocatechuic acid	Flavanols	0.6	0.4	0.9	−0.2	0.3	0.5
pmb0736	4.7	492.1	C_23_H_24_O_12_	[M+H]^+^	Tricin 7-O-hexoside	Flavonoid	0.3	0.0	−6.3	−0.3	−6.6	−6.4
Li512117	4.4	610.1	C_27_H_30_O_16_	[M+H]^+^	Quercetin 3-O-rhanosylgalactoside	Flavonols	1.6	2.1	2.2	0.5	0.6	0.1
GQ512006	4.1	610.1	C_27_H_30_O_16_	[M+H]^+^	Quercetin-3-O-glucoside-7-O-rhamnoside	Flavonols	1.6	2.1	2.2	0.5	0.6	0.1
pme2493	4.3	578.1	C_27_H_30_O_14_	[M+H]^+^	Kaempferol 3,7-dirhamnoside(Kaempferitrin)	Flavonols	0.6	0.5	1.2	−0.2	0.6	0.8
pmb0665	4.2	610.1	C_27_H_30_O_16_	[M+H]^+^	Luteolin 8-C-hexosyl-O-hexoside	Flavonoid	1.3	2.0	2.0	0.6	0.7	0.1
pmn001583	4.1	610.1	C_27_H_30_O_16_	[M-H]^−^	Bioquercetin	Flavonols	1.3	2.0	2.0	0.7	0.7	0.0
pmp000548	6.4	300.1	C_16_H_12_O_6_	[M+H]^+^	Pratensein	Isoflavones	4.9	3.7	2.4	−1.3	−2.5	−1.3
pmp001127	6.7	300.1	C_16_H_12_O_6_	[M+H]^+^	Chrysoeriol	Flavonoid	4.9	3.6	2.4	−1.3	−2.5	−1.2
Zmjp003072	4.3	610.1	C_30_H_26_O_14_	[M+H]^+^	Quercetin-p-coumaroyl hexose	Flavonoids	1.5	2.0	2.3	0.6	0.8	0.2
pmb3023	3.8	450.1	C_21_H_22_O_11_	[M–H]^−^	Eriodictyol C-hexoside	Dihydroflavone	−2.2	−1.1	−1.6	1.1	0.6	−0.5
mws1397	4.3	442.1	C_22_H_18_O_10_	[M–H]^−^	(–)-Epicatechin gallate	Flavanols	−0.2	−1.9	−0.5	−1.7	−0.3	1.4
mws0355	4.2	442.1	C_22_H_18_O_10_	[M–H]^−^	(–)-Catechin gallate	Flavanols	−0.2	−2.0	−0.4	−1.8	−0.2	1.5

### Integration of the Transcriptome and Metabolome Profiles Related to Flavonoid Biosynthesis

**Figure 3:**
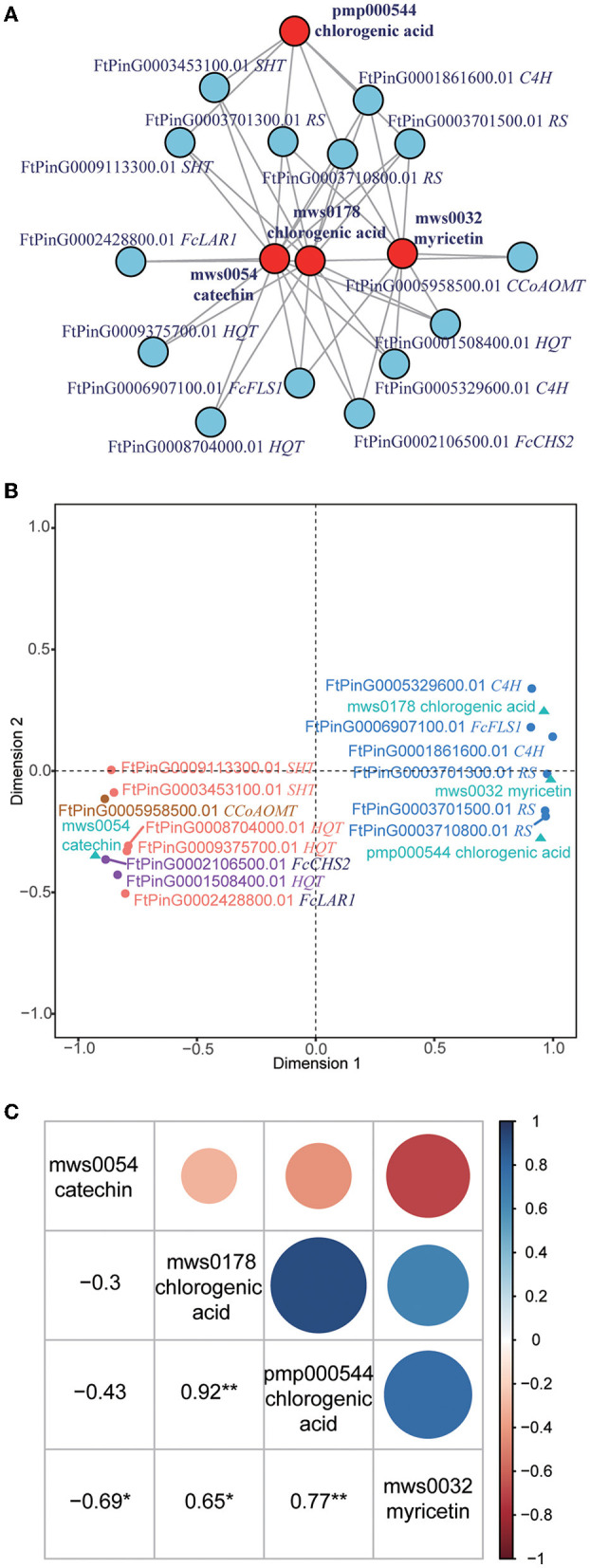
Integration of the transcriptome and metabolome profiles. **(A)** Interaction network of metabolites and genes related to catechin. The metabolites are presented as red circles, and the genes are presented as blue circles. CHS, chalcone synthases; HQT, hydroxycinnamoyl-coenzyme A quinate transferase; SHT, spermidine hydroxycinnamoyl transferase; RS, resveratrol synthase; C4H, cinnamate 4-hydroxylase; LAR, leucoanthocyanidin reductase; FLS, flavonol synthase; CCoAOMT, caffeoyl-CoA O-methyltransferase. **(B)** Canonical correlation analysis (CCA) of metabolites and genes related to catechin. The metabolites are presented as green triangles. Genes in K1 are presented as purple dots. Genes in K2 are presented as orange dots. Genes in K3 are presented as blue dots. Genes in K4 are presented as brown dots. **(C)** Pearson correlation of four metabolites in the related network. *Indicates that the correlation reached a significant level of 0.05. **Indicates that the correlation reached a significant level of 0.01.

Integration analysis of the transcriptome and metabolome profiles revealed tens of thousands of correlations between genes and metabolites. What we focused on was the correlations in flavonoid biosynthesis (KEGG pathway: ko00941), in which a total of 32 genes were correlated to 15 metabolites (Supplementary Table 6). As catechin is the most abundant flavonoid, we subsequently examined whether some genes or metabolites interacted with each other. Interestingly, we found that 14 genes interacted with four metabolites, namely, catechin (mws0054), myricetin (mws0032), and two chlorogenic acids (mws0178 and pmp000544) (Figure 3A). Genes in this interaction network included 1 *CHS* gene *(FcCHS2)*, 3 hydroxycinnamoyl-coenzyme A quinate transperases (HQT, FtPinG0001508400.01, FtPinG0009375700.01, and FtPinG0008704000.01), 2 spermidine hydroxycinnamoyl transferases (SHT, FtPinG0003453100.01 and FtPinG0009113300.01), 3 resveratrol synthases (RS, FtPinG0003701300.01, FtPinG0003701500.01, and FtPinG0003710800.01), 2 *C4H* genes (FtPinG0001861600.01 and FtPinG0005329600.01), 1 *LAR* gene (FcLAR2), 1 *FLS* gene (FtPinG0006907100.01), and one caffeoyl-CoA O-methyltransferase (CCoAOMT, FtPinG0005958500.01).

Based on their correlation with the metabolites, the 14 genes were divided into four classes (K1 to K4) based on CCA analysis (Figure 3B). K1 included two genes (*FcCHS2* and FtPinG0001508400.01) that were positively correlated to catechin but negatively correlated to myricetin and chlorogenic acids. K2 included five genes (FtPinG0009375700.01, FtPinG0008704000.01, FtPinG0003453100.01, FtPinG0009113300.01, and *FcLAR2*) that were positively correlated to catechin but negatively correlated to chlorogenic acids. K3 included six genes (FtPinG0003710800.01, FtPinG0006907100.01, FtPinG0003701500.01, FtPinG0001861600.01, FtPinG0005329600.01, and FtPinG0003701300.01) that were negatively correlated to catechin but positively correlated to myricetin and chlorogenic acids. K4 included one gene (FtPinG0005958500.01) that was positively correlated to myricetin and chlorogenic acids.

The Pearson correlation of four metabolites in the same network was also analyzed (Figure 3C). Notably, catechin (mws0054) was significantly negatively correlated to myricetin (mws0032) (*R* = −0.69). Myricetin (mws0032) was significantly positively correlated to two chlorogenic acids (mws0178 and pmp000544) (*R* = 0.65 and 0.77) but negatively correlated to catechin (mws0054). The correlation of two chlorogenic acids (mws0178 and pmp000544) was highest, with a Pearson R-value of 0.92.

### MYB Transcription Factor Regulated Flavonoid Biosynthesis

**Figure 4:**
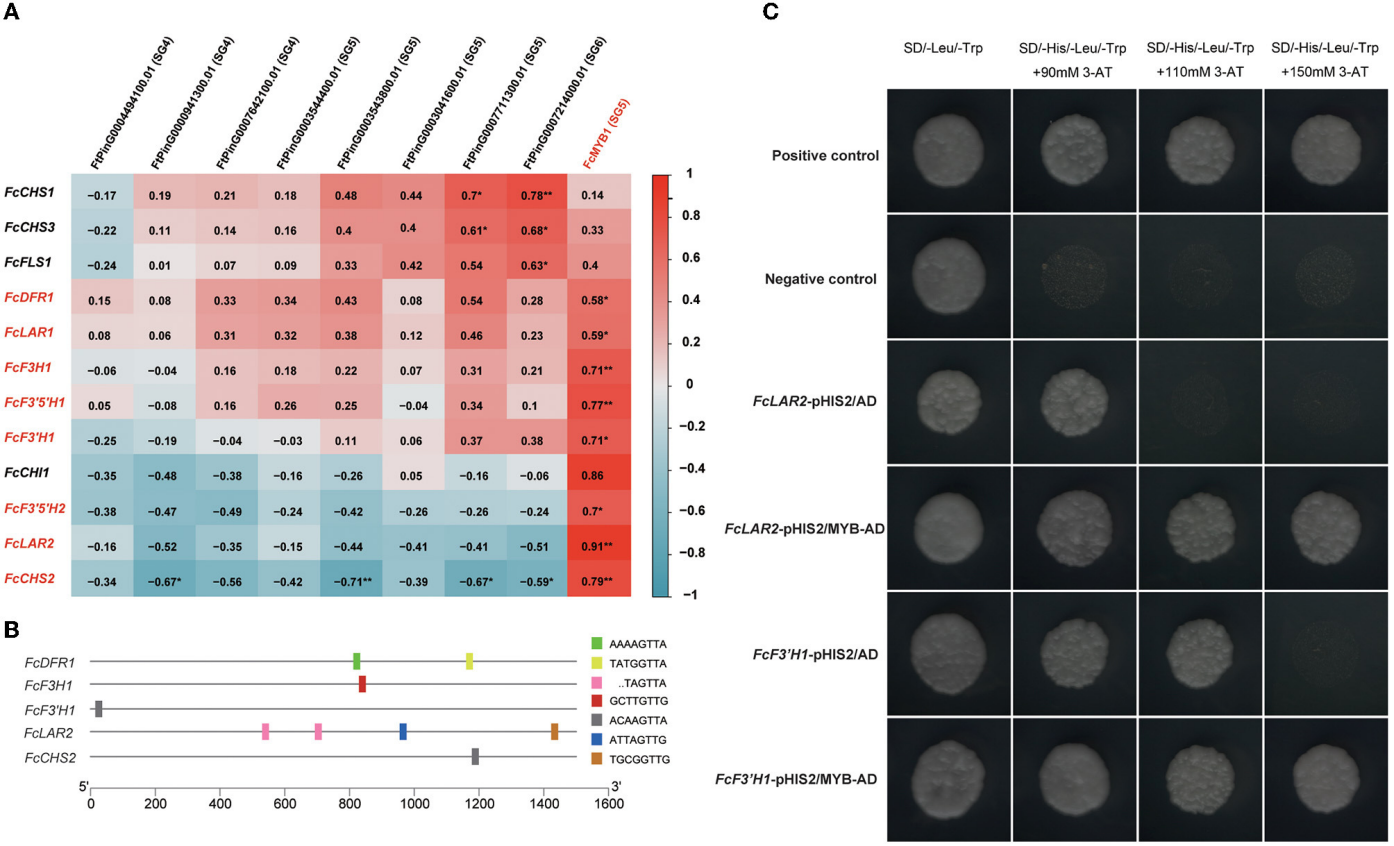
MYB transcription factor (TF) regulated flavonoid biosynthesis. **(A)** Pearson correlations of flavonoid biosynthesis DEGs and MYB DEGs. **(B)** DNA-binding specificities of R2R3-MYB TFs identified in the promoters of flavonoid biosynthesis DEGs. **(C)** Yeast one-hybrid analysis of FcMYB1 to examine the binding of the protein to the promoters of flavonoid biosynthesis DEGs.

It is widely reported that R2R3-MYB transcription factors play essential roles in the regulation of flavonoid biosynthesis (Xu et al., 2015). In Tartary buckwheat, SG4, SG5, SG6, SG7 subfamily MYBs are responsible for this process (Zhang et al., 2017, 2018, 2019; Li et al., 2019c; Yao et al., 2020; Ding et al., 2021). In order to learn more about the genes in the regulatory network of flavonoid biosynthesis, we collected all DEGs annotated to flavonoid biosynthesis genes and SG4, SG5, SG6, SG7 subfamily MYBs and resulting in 12 flavonoid biosynthesis DEGs and nine MYB DEGs (Figure 4A). Pearson correlations of the flavonoid biosynthesis DEGs and MYB DEGs were calculated. The results showed that an SG5 subfamily MYB, *FcMYB1*, was positively correlated to eight flavonoid biosynthesis genes, including two *LAR* homologs (*FcLAR2*, Pearson *R* = 0.91, and *FcLAR1*, Pearson *R* = 0.59), a *DFR* homolog (*FcDFR1*, Pearson *R* = 0.58), an *F3H* homolog (*FcF3H1*, Pearson *R* = 0.71), two *F3*′*5*′*H* homologs (*FcF3*′*5*′*H1*, Pearson *R* = 0.77, and *FcF3*′*5*′*H2*, Pearson *R* = 0.7), an *F3*′*H* homolog (*FcF3*′*H1*, Pearson *R* = 0.71), and a *CHS* homolog (*FcCHS2*, Pearson *R* = 0.79) (Figure 4A). The highly correlated expression patterns suggested that *FcMYB1* might be an essential regulator in flavonoid biosynthesis.

Previous studies have exhaustively characterized the DNA-binding specificities of R2R3-MYB proteins in relation to flavonoid biosynthesis. Nine DNA-binding specificities could bind to most R2R3-MYB proteins related to flavonoid biosynthesis in plants, namely, TACTGTTG, TGCGGTTG, AAAAGTTA, GTCAGTTA, ACAAGTTA, ATTAGTTG, GCTTGTTG, TATGGTTA, and TAGTTA (Kelemen et al., 2015). Therefore, we obtained and scanned the 1,500 bp promoter sequences from the translation initiation site of the eight DEGs in flavonoid biosynthesis that were highly correlated to *FcMYB1* to determine whether these DNA-binding specificities were included in them. Notably, *FcLAR2* had four R2R3-MYB DNA-binding specificities on its promoter; *FcDFR1* had two R2R3-MYB DNA-binding specificities on its promoter; *FcCHS2, FcF3*′*H1*, and *FcF3H1* had one R2R3-MYB DNA-binding specificity on each of their promoters (Figure 4B). These suggested that *FcMYB1* might directly bind to the promoter of these five genes to regulate flavonoid biosynthesis.

Yeast one-hybrid technology was performed to determine the interactions of the FcMYB1 protein and the promoter of five flavonoid biosynthesis genes, namely, *FcCHS2, FcLAR2, FcDFR1, FcF3*′*H1*, and *FcF3H1*. The constructs of pHIS2-promoter and pGADT7-MYB were co-transformed into yeast strain Y187. The results showed that MYB could bind to the promoter of *FcLAR2* and *FcF3*′*H1* (Figure 4C) but could not bind to the promoter of the other three genes. This suggested that MYB had a DNA-binding activity and could directly regulate the expression of *FcLAR2* and *FcF3*′*H1*.

### Validation of Gene Expression by qRT-PCR Analysis

**Figure 5:**
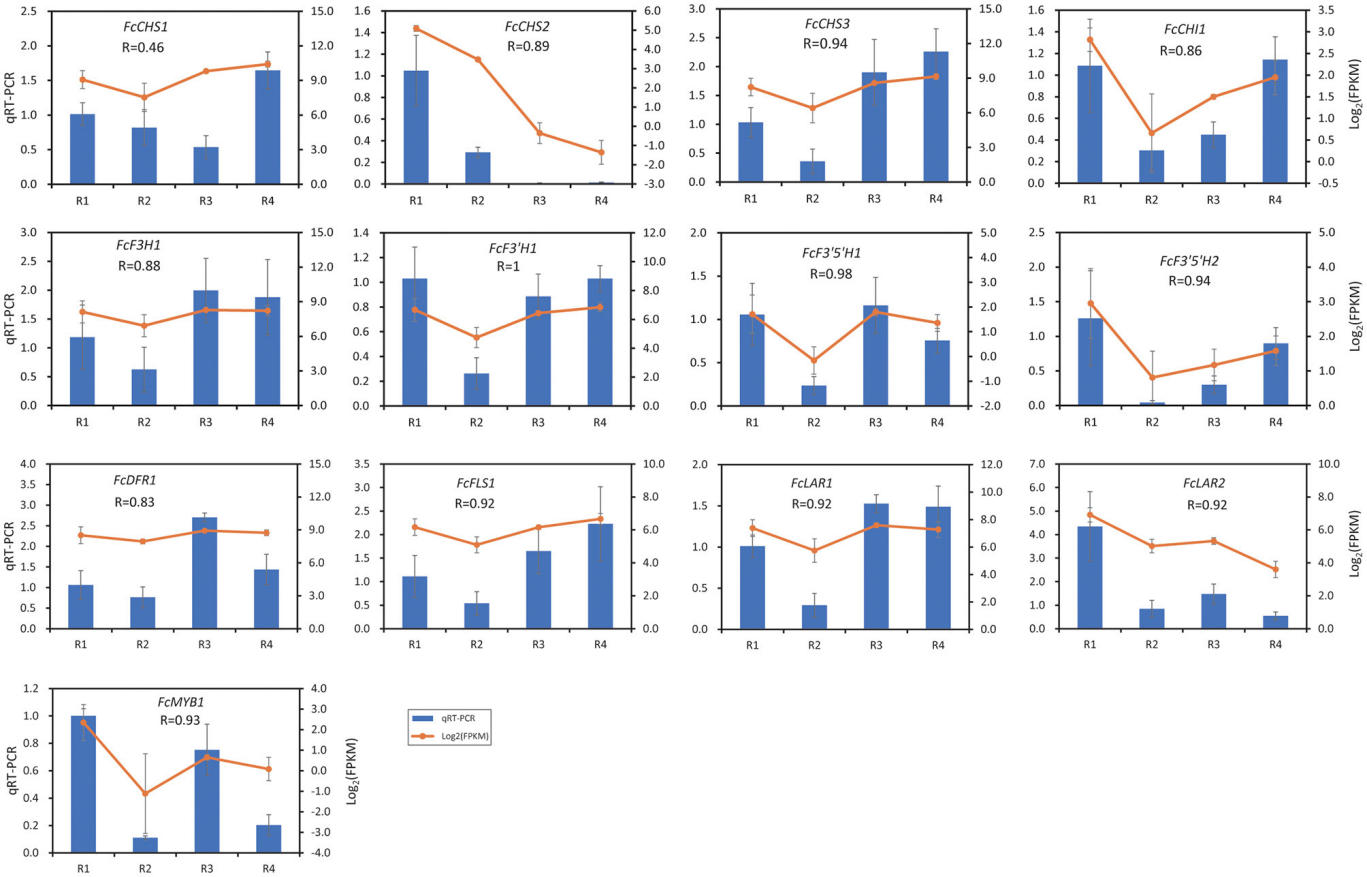
Validation of gene expression by qRT-PCR analysis. The value of data is the average expression value of the transcriptome and qRT-PCR data. The error bar represents the standard deviation.

The expression of 13 DEGs was validated by qRT-PCR, in which 12 flavonoid biosynthesis DEGs and *FcMYB1* that was mentioned in Figure 4 were included. As shown in Figure 5, the expressions of 12 out of the 13 DEGs were well correlated by qRT-PCR, with a Pearson R ranging from 0.83 to 1. The Pearson R of *FcMYB1* was 0.93. The Pearson R of *FcLAR2* and *FcF3*′*H1*, whose promoter could be bound by the FcMYB1protein, was 0.92 and 1, respectively. One CHS gene, namely, *FcCHS1*, showed a lower correlation between the transcriptome and qRT-PCR (Pearson *R* = 0.46), so it was eliminated in the subsequent analysis.

### Flavonoid Biosynthesis Pathway in the Rhizomes of Golden Buckwheat

**Figure 6:**
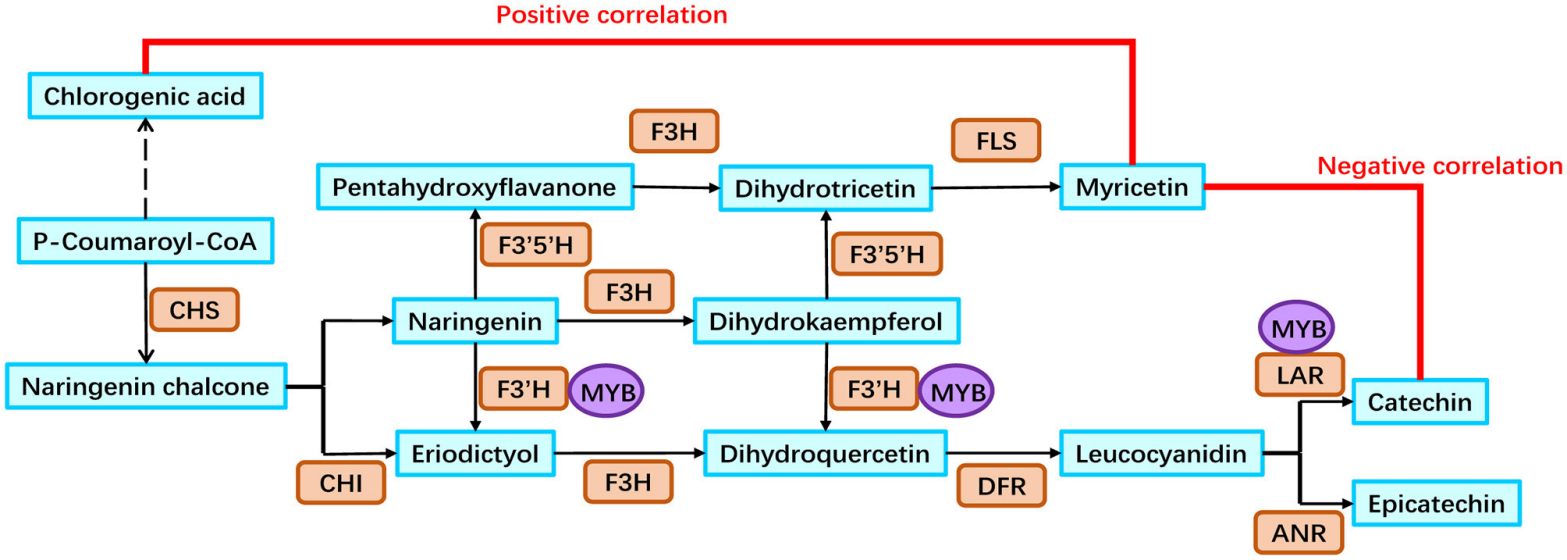
Flavonoid biosynthesis pathway in the rhizomes of golden buckwheat. Cyan boxes represent the metabolites in the flavonoid biosynthesis pathway. Orange rounded boxes represent enzymes that catalyze the reactions in the flavonoid biosynthesis pathway. Purple circles represent the FcMYB1 protein that could bind to the promoters of candidate genes. Gene names of 11 enzymes are listed as follows: *FcCHS2, FcCHS3, FcCHI1, FcF3H1, FcF3*′*H1, FcF3*′*5*′*H1, FcF3*′*5*′*H2, FcDFR1, FcFLS1, FcLAR1*, and *FcLAR2*.

Based on our analyses, a putative flavonoid biosynthesis pathway in the rhizomes of golden buckwheat was proposed (Figure 6). This included 13 metabolites, 11 genes that were annotated to encode eight enzymes, and one MYB transcription factor. The expression patterns of genes in this pathway are shown in Figure 5 and validated by qRT-PCR.

## Discussion

### First Integrative Transcriptomics and Metabolomics Analyses of Golden Buckwheat

Among the three buckwheat species, Tartary buckwheat is the most thoroughly studied species in molecular biology. This is largely due to its high content of flavonoids, which are known as antioxidants with anti-inflammatory, anticancer, and vascular-protective properties (Fabjan et al., 2003). Since its genome sequencing was finished years ago, flavonoid biosynthesis and its regulatory mechanisms have been extensively reported (Zhang et al., 2017, 2019; Li et al., 2019c; Yao et al., 2020; Ding et al., 2021). Meanwhile, types and amounts of Tartary buckwheat flavonoids were identified and measured (Fabjan et al., 2003; Ma et al., 2019; Li et al., 2019a,b; Yang et al., 2020; Hou et al., 2021). By contrast, only a few studies related to the above areas were reported on golden buckwheat, as its genome is complex and not sequenced yet, which limited its molecular biology development. To date, only one transcriptome analysis of underlying proanthocyanidin accumulation was performed by *de novo* assembly, and eight coding sequences in flavonoid biosynthesis were cloned in golden buckwheat (Liu et al., 2009; Ma et al., 2009, 2012; Meng et al., 2010; Li et al., 2011; Jiang et al., 2013; Luo et al., 2013; Pu et al., 2014; Chen and Li, 2016). In the current transcriptome, we tried to map the clean reads to Tartary buckwheat genome data, in view of *F. tataricum* being very close to *F. cymosum* (Ohnishi and Matsuoka, 1996), which showed the expected results, with a unique mapping percentage ranging from 63.6–72.36%. A total of 28,492 genes based on the genome data of Tartary buckwheat and 1,665 new genes not included in the reference genome data were identified in the rhizomes of golden buckwheat, among which 10,191 genes were DEGs. This would enrich the gene sequence resources of golden buckwheat.

According to the metabolome analysis, 439 metabolites were identified, including 90 flavonoids. The number of flavonoids in golden buckwheat was much less than that in Tartary buckwheat (Ma et al., 2019; Zhao et al., 2019; Li et al., 2019a,b). A recent study determined nine flavonoids from rhizomes of golden buckwheat, namely, catechin, epicatechin, epicatechin gallate, gallocatechin, epigallocatechin, catechin gallate, epicatechin gallate, afzelechin, and epiafzelechin, of which catechin and epicatechin together constituted about 90% (Li et al., 2021). Compared with our study, eight of the nine flavonoids were included in our metabolome except for afzelechin. All the other 82 flavonoids were identified in golden buckwheat for the first time. Catechin, gallic acid, hesperidin, nepetin, and L-epicatechin were the top five flavonoids identified in this study, which together constituted about 57% of the total flavonoids. This difference in the percentage of catechin and epicatechin is largely due to the difference in methods. Our metabolome was a large-scale method that included more than 1,000 flavonoids in the constructed flavonoid metabolite database (Chen et al., 2013). The most abundant flavonoid in Tartary buckwheat was rutin, which belongs to the flavonol subclass and accounts for 50–80% of total flavonoids (Fabjan et al., 2003; Li et al., 2019a; Ma et al., 2019). In our golden buckwheat metabolome data, rutin was 23rd.

To our knowledge, this is the first metabolome study, and the first to integrate metabolome and transcriptome data in golden buckwheat. Based on our analyses, we enriched the expressed genes and the existing metabolites and proposed a flavonoid biosynthesis pathway in golden buckwheat rhizomes.

### Integration Analysis of the Transcriptome and Metabolome Revealed a Catechin Integrative Network in the Rhizomes of Golden Buckwheat

A catechin integrative network was identified based on the transcriptome and metabolome integration analyses, in which 14 genes and four metabolites were included. The Pearson correlation of four metabolites indicated that catechin (mws0054) was negatively correlated to myricetin (mws0032) and chlorogenic acids (mws0178 and pmp000544), which implied two competitive synthesis reactions during catechin biosynthesis. The first competitive synthesis reaction occurred during the biosynthesis of chlorogenic acids and flavonoids where they competed for the same substrate, p-coumaroyl-CoA. The formation of p-coumaroyl-CoA by 4CL was a common step for both chlorogenic acid biosynthesis and flavonoid biosynthesis (Kim et al., 2013; Saito et al., 2013; Tohge et al., 2017). After this step, HQT catalyzed p-coumaroyl-CoA to p-coumaroyl quinic acid or hydroxycinnamoyl-CoA shikimate/quinate hydroxycinnamoyl transferase catalyzed p-coumaroyl-CoA to p-coumaroyl shikimic, which started the biosynthesis pathway of chlorogenic acids (Kim et al., 2013). Meanwhile, CHS catalyzed p-coumaroyl-CoA to naringenin chalcone, which started the biosynthesis pathway of flavonoids (Saito et al., 2013; Tohge et al., 2017). Catechin was the most abundant flavonoid, so it was negatively correlated to chlorogenic acids. The second competitive synthesis reaction occurred during the biosynthesis of catechin and myricetin. Although catechin is a kind of flavanol and myricetin is a flavonol, naringenin and dihydrokaempferol are their common upstream substrates. For catechin biosynthesis, naringenin and dihydrokaempferol were first catalyzed by F3′H to form dihydroquercetin; then dihydroquercetin was catalyzed by DFR to form leucocyanidin; finally, leucocyanidin was catalyzed by LAR to form catechin. For myricetin biosynthesis, naringenin and dihydrokaempferol were first catalyzed by F3′5′H to form dihydrotricetin; then, dihydrotricetin was catalyzed by FLS to form myricetin (Saito et al., 2013; Tohge et al., 2017). They competed with the same substrates during their biosynthesis, so they were negatively correlated to each other.

It is worth mentioning that the enzymes in the network were proposed based on the gene expression in transcriptome data. Although there are some reports that construct the flavonoid biosynthesis pathway based on transcriptome data (Chen and Li, 2016; Chen et al., 2020; Zhang et al., 2022), we do not have experimental evidence for the enzymatic activity of gene products. Therefore, enzyme activity should be determined for further analyses.

### MYB Transcription Factor Positively Regulated Catechin Biosynthesis

In plants, R2R3-MYB transcription factors are involved in the regulation of flavonoid biosynthesis by binding to DNA-binding specificities on promoters of flavonoid biosynthesis genes (Kelemen et al., 2015; Xu et al., 2015). In Tartary buckwheat, massive genes have been identified and, thus, form a comprehensive flavonoid biosynthesis pathway, in which some R2R3-MYB transcription factors are included (Zhang et al., 2017, 2018, 2019; Li et al., 2019c; Yao et al., 2020; Ding et al., 2021). After the release of the complete Tartary buckwheat genome, genes involved in rutin biosynthesis and regulation pathway were comprehensively identified (Zhang et al., 2017). Afterward, several FtMYBs were found to directly regulate flavonoid biosynthesis, among which FtMYB11, FtMYB13, FtMYB14, FtMYB15, and FtMYB16 could directly bind to the MBS site on the promoter of *FtPAL* and repress rutin biosynthesis, FtMYB116 could bind directly to the promoter region of *FtF3*′*H* and increase the content of rutin and quercetin, and FtMYB6 promoted the activity of *FtF3H* and *FtFLS1* promoters and significantly increased the accumulation of flavonols (Zhang et al., 2018, 2019; Li et al., 2019c; Yao et al., 2020; Ding et al., 2021). However, there has been no reported regulator involved in the flavonoid biosynthesis of golden buckwheat so far. We identified a novel MYB transcription factor, *FcMYB1*, whose expression patterns were positively correlated to eight out of 12 flavonoid biosynthesis genes significantly. The Y1H analysis showed that the FcMYB1 protein could directly bind to the promoters of *FcLAR2* and *FcF3*′*H1*. F3′H is an essential enzyme that promotes the formation of flavanols in the competitive biosynthesis of flavonols and flavanols, and LAR is the key enzyme catalyzing leucocyanidin to form catechin (Saito et al., 2013; Tohge et al., 2017). This suggested that FcMYB1 might be a transcriptional activator in catechin biosynthesis by directly binding to the promoters of *FcLAR2* and *FcF3*′*H1*. This is the first identified regulator in the flavonoid biosynthesis of golden buckwheat. It is a very good beginning for further identification of flavonoid regulatory factors including R2R3-MYB, bHLH, WD40 proteins, and the interaction of MYB-bHLH-WD40 complex in golden buckwheat.

## Conclusion

In conclusion, for the first time, this study performed transcriptome and widely targeted metabolome analyses using four golden buckwheat accessions that differed in total flavonoid content. A total of 10,191 DEGs and 297 DRMs were identified, among which the flavonoid biosynthesis pathway was enriched in both transcriptome and metabolome. Integration analyses of the transcriptome and metabolome profiles revealed a network related to catechin in which 4 metabolites and 14 genes interacted with each other. An SG5 R2R3-MYB transcription factor, *FcMYB1*, was found as a transcriptional activator in catechin biosynthesis for the first time, as it was positively correlated to eight flavonoid biosynthesis genes in their expression patterns and could directly bind to the promoters of *FcLAR2* and *FcF3*′*H1*. Finally, a flavonoid biosynthesis pathway in the rhizomes of golden buckwheat was proposed, including 13 metabolites, 11 genes encoding 9 enzymes, and 1 MYB transcription factor. The result provided a comprehensive flavonoid biosynthesis and regulatory network of golden buckwheat.

## Data Availability Statement

The raw sequence data are deposited in the Genome Sequence Archive in the National Genomics Data Center, Chinese Academy of Sciences, under accession number CRA003372 which is publicly accessible at https://ngdc.cncb.ac.cn/gsa.

## Author Contributions

JH and QC designed and coordinated the study. LW, BT, RR, TS, LZ, and CL planted the materials, took the samples, and carried out the experiments. JH, JD, and LW analyzed the transcriptomics and metabolomics data, made the figures and wrote the draft of the manuscript. All authors approved the final version of the manuscript.

## Funding

This research was funded by the Joint Project of the National Science Foundation of China and Guizhou Provincial Government Karst Science Research Center (U1812401), National Natural Science Foundation of China (32060508), Science and Technology Foundation of Guizhou Province (QianKeHeJiChu-ZK[2021] General 109), and National Key Research and Development Program of China (2019YFD1001300/2019YFD1001304).

## Conflict of Interest

The authors declare that the research was conducted in the absence of any commercial or financial relationships that could be construed as a potential conflict of interest.

## Publisher's Note

All claims expressed in this article are solely those of the authors and do not necessarily represent those of their affiliated organizations, or those of the publisher, the editors and the reviewers. Any product that may be evaluated in this article, or claim that may be made by its manufacturer, is not guaranteed or endorsed by the publisher.

## Supplementary Material

Supplementary Figure 1 Sample cluster of the transcriptome. Each square indicates the Pearson's correlation coefficient of a pair of samples.

Supplementary Figure 2 Top 30 enriched Gene Ontology (GO) items of the transcriptome. The results are summarized in three main categories: biological process, cellular component, and molecular function.

Supplementary Figure 3 Top 20 enriched Kyoto Encyclopedia of Genes and Genomes (KEGG) pathways of the transcriptome. The rich factor is the ratio of the number of DEGs to that of all genes annotated to a pathway term. A higher rich factor indicates greater intensity.

Supplementary Figure 4 PCA analysis of the metabolome.

Supplementary Figure 5 K means a cluster of the differentially regulated metabolites. The x-axis corresponds to the samples and the y-axis to standardized intensity.

Supplementary Figure 6 Top 20 enriched KEGG pathways of the metabolome. The rich factor is the ratio of the number of DEGs to that of all genes annotated to a pathway term. A higher rich factor indicates greater intensity.

Supplementary Table 1 List of all the primers used in this study.

Supplementary Table 2 Summary statistics of RNA sequencing results of golden buckwheat rhizomes.

Supplementary Table 3 Differentially expressed genes (DEGs) identified among the four golden buckwheat rhizomes. DESeq2 was used to identify the DEGs, and thresholds were set as the absolute value of log_2_ratio ≥ 1 and *P*-value (padj) <0.05.

Supplementary Table 4 All metabolites identified in golden buckwheat rhizomes.

Supplementary Table 5 DRMs identified in golden buckwheat rhizomes. MetaboAnalystR was used to identify the DRMs by variable importance in the projection value (VIP) ≥ 1 and absolute log_2_ratio ≥ 1.

Supplementary Table 6 Correlations between genes and metabolites in flavonoid biosynthesis (KEGG pathway ko00941).
