# Supplementary figures and images for: Integrated Transcriptomics and Widely Targeted Metabolomics Analyses Provide Insights Into Flavonoid Biosynthesis in the Rhizomes of Golden Buckwheat (Fagopyrum cymosum)
Source: Front Plant Sci. 2022 Jun 17;13:803472. doi: 10.3389/fpls.2022.803472 (PMC9247553; doi:10.3389/fpls.2022.803472)

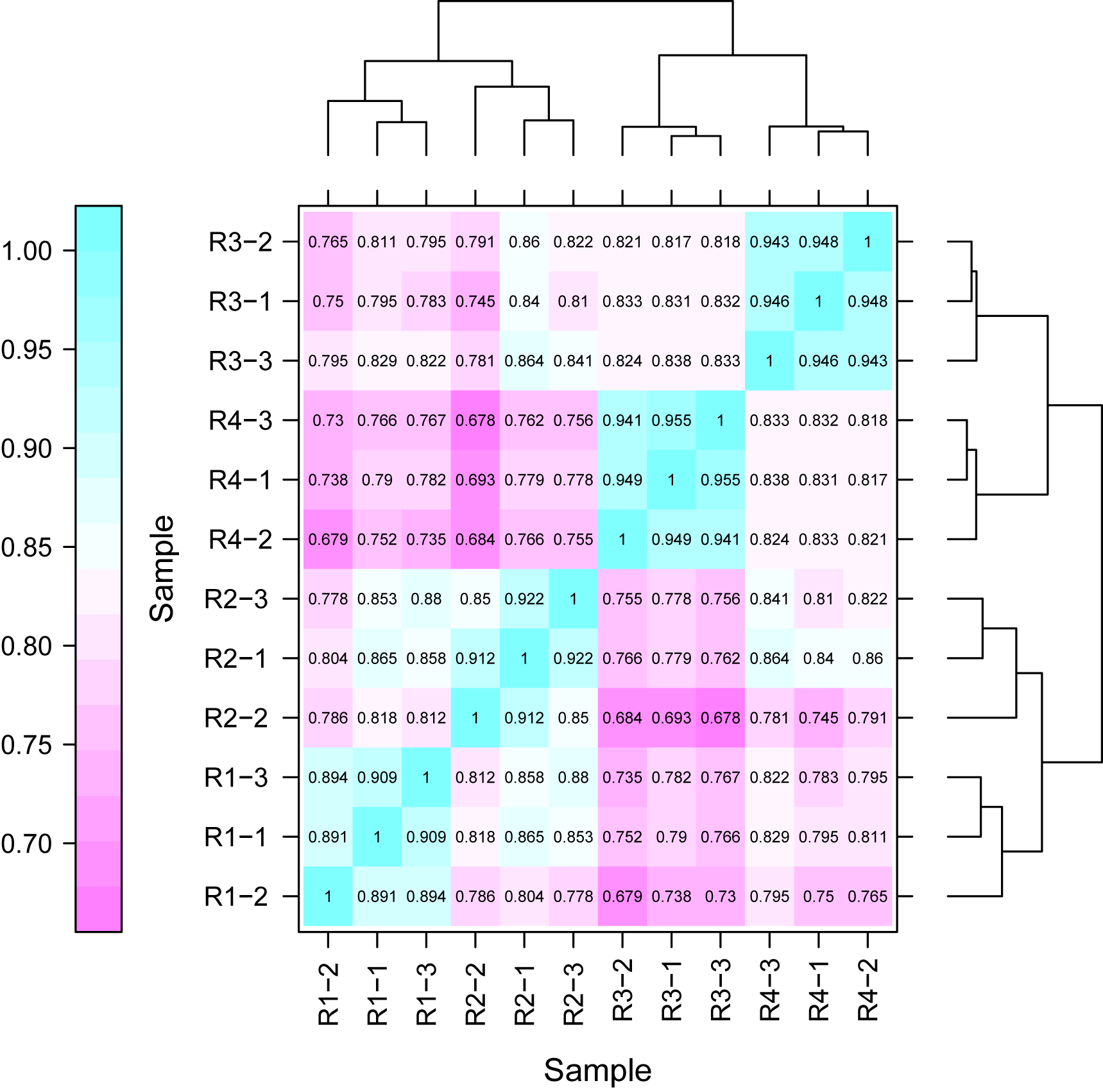

Supplement: Supplementary Figure 1 — Sample cluster of the transcriptome. Each square indicates the Pearson's correlation coefficient of a pair of samples. [file Image_1.TIF]

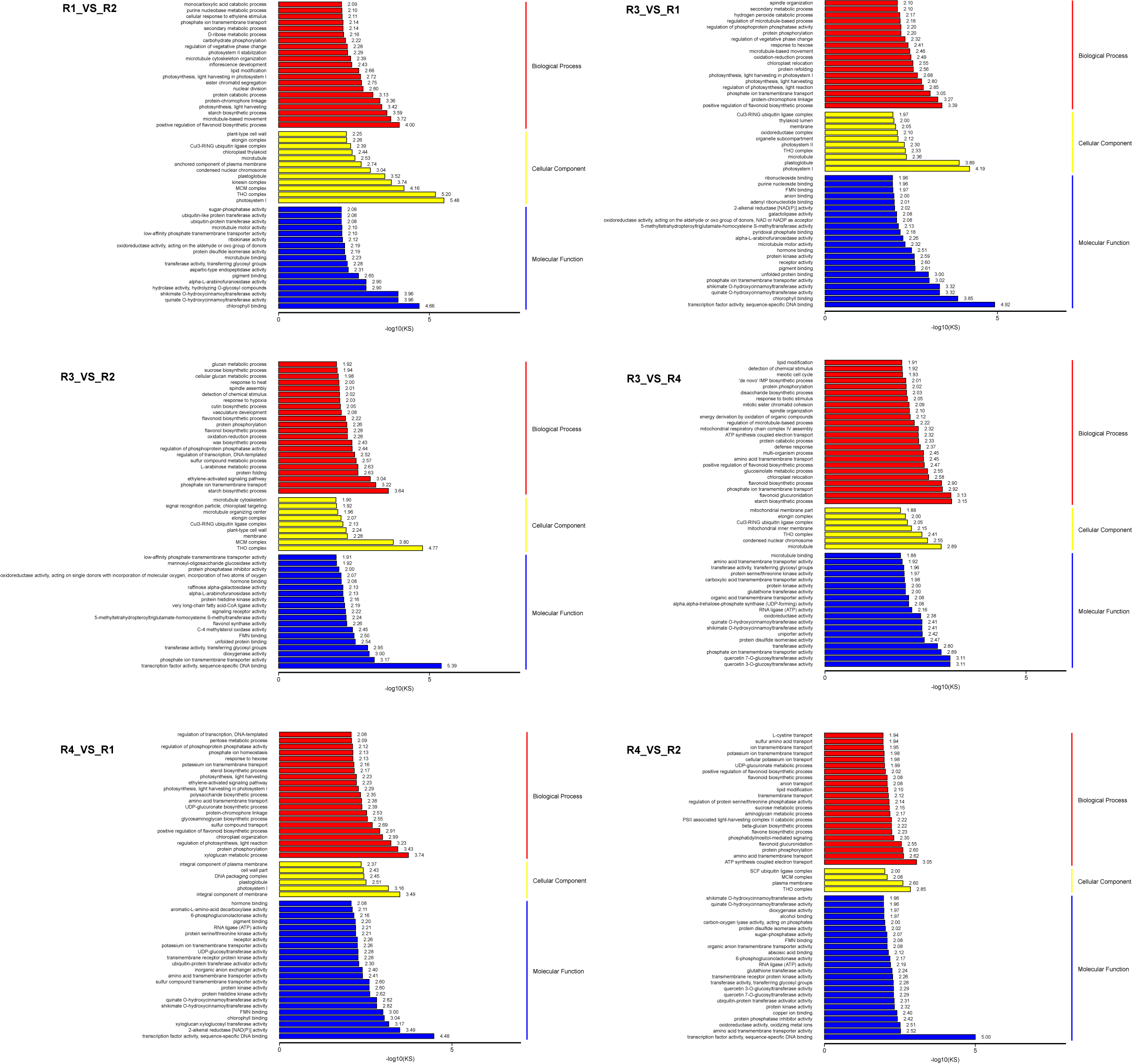

Supplement: Supplementary Figure 2 — Top 30 enriched Gene Ontology (GO) items of the transcriptome. The results are summarized in three main categories: biological process, cellular component, and molecular function. [file Image_2.TIF]

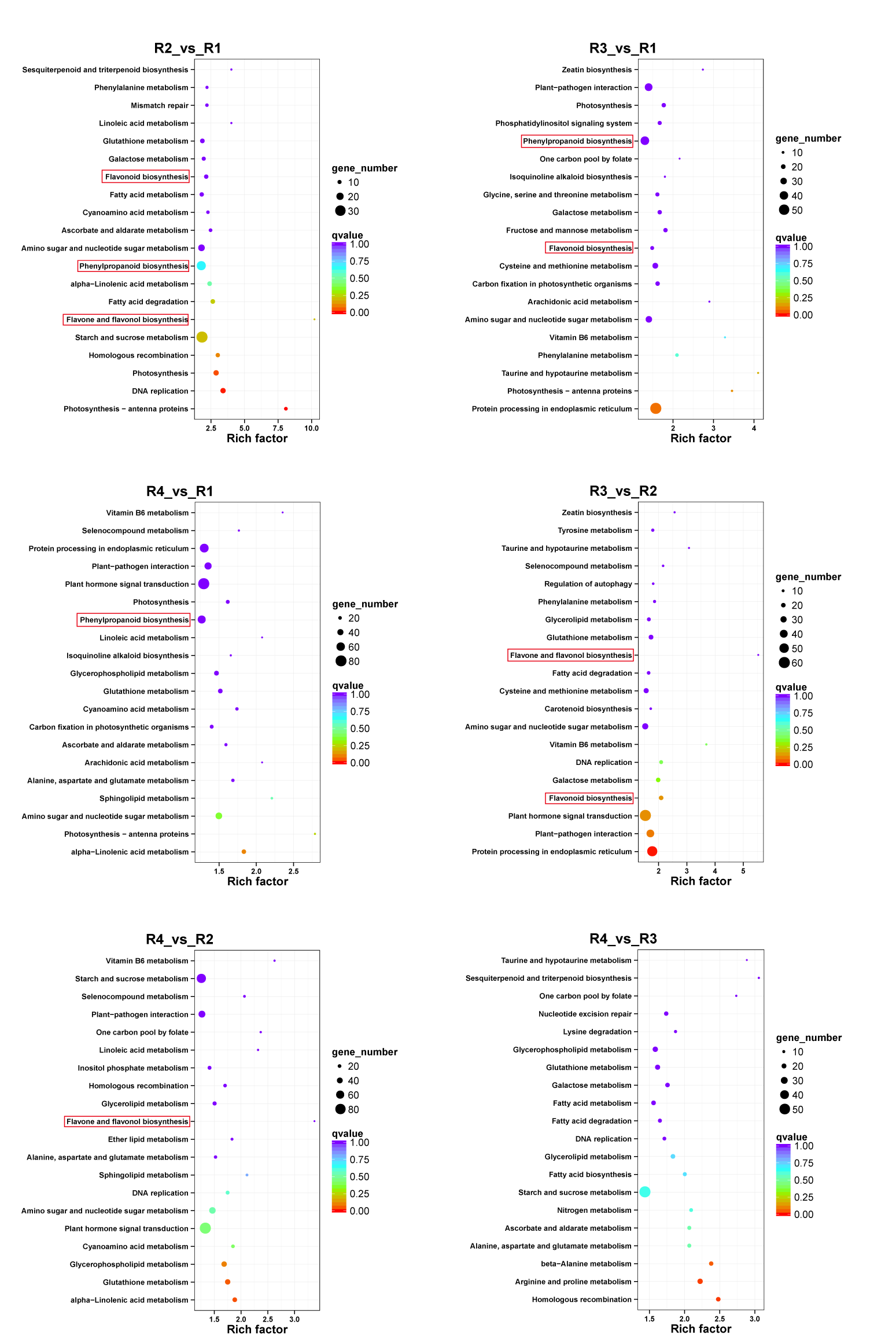

Supplement: Supplementary Figure 3 — Top 20 enriched Kyoto Encyclopedia of Genes and Genomes (KEGG) pathways of the transcriptome. The rich factor is the ratio of the number of DEGs to that of all genes annotated to a pathway term. A higher rich factor indicates greater intensity. [file Image_3.TIF]

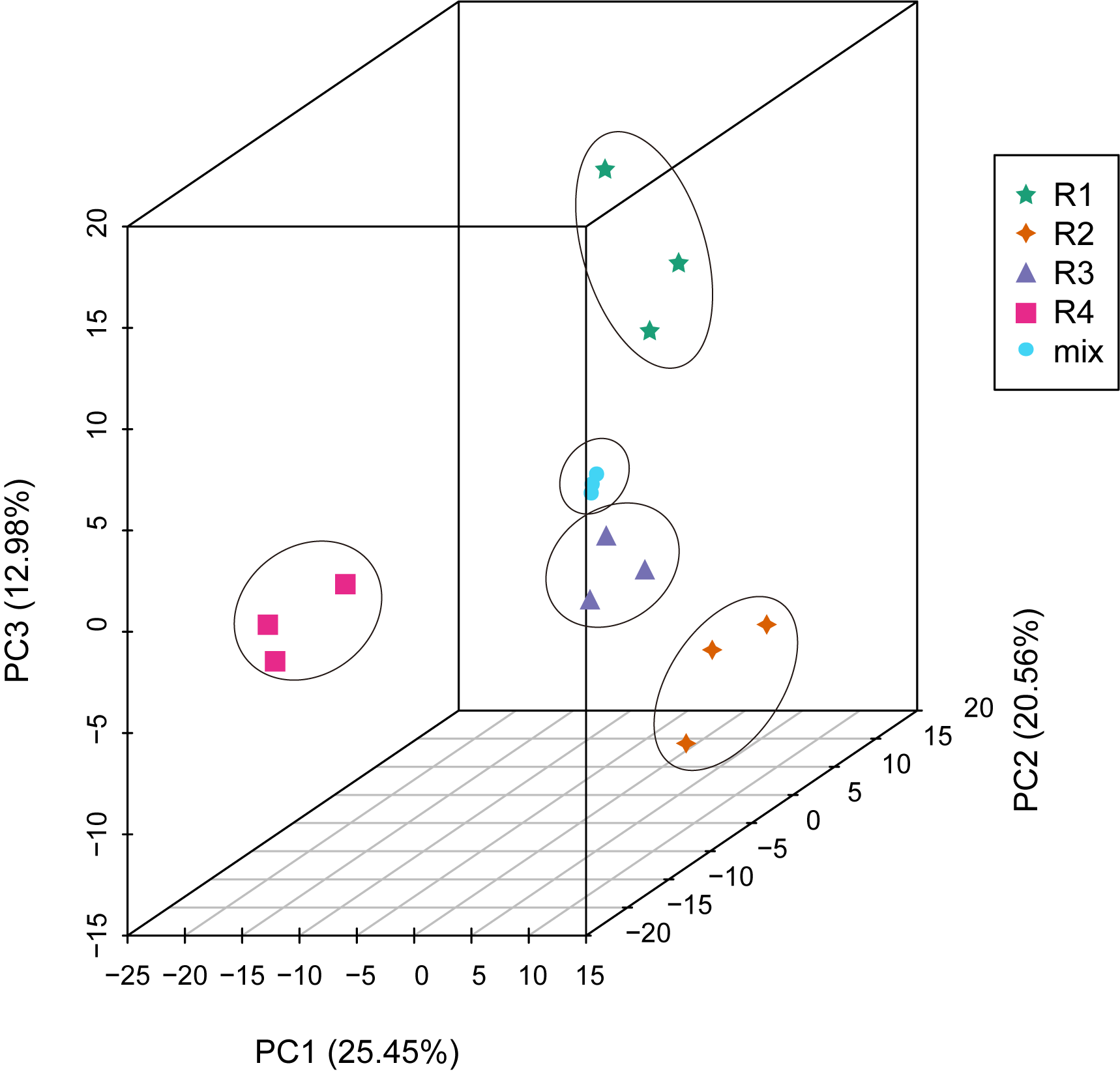

Supplement: Supplementary Figure 4 — PCA analysis of the metabolome. [file Image_4.TIF]

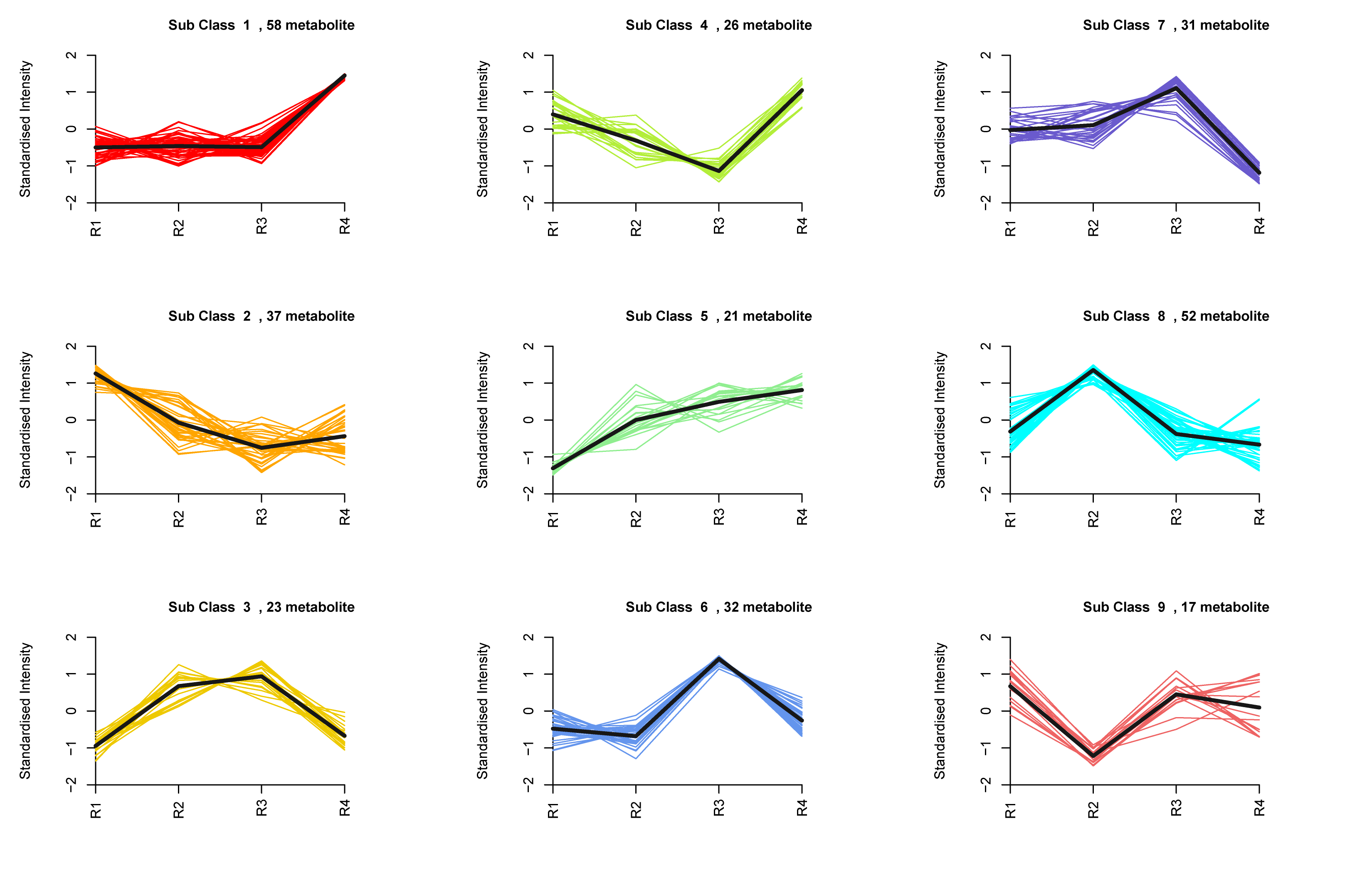

Supplement: Supplementary Figure 5 — K means a cluster of the differentially regulated metabolites. The x-axis corresponds to the samples and the y-axis to standardized intensity. [file Image_5.TIF]

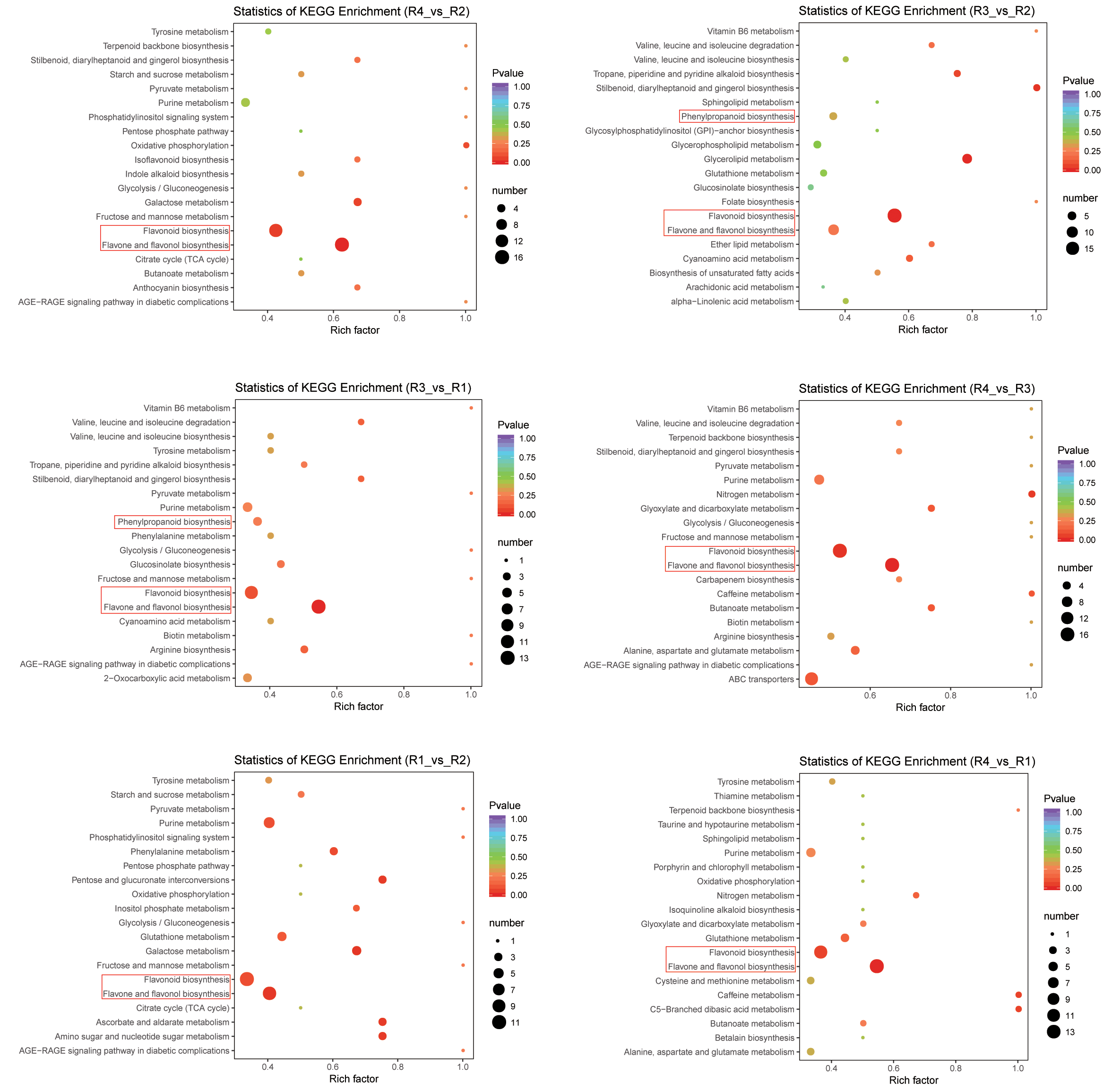

Supplement: Supplementary Figure 6 — Top 20 enriched KEGG pathways of the metabolome. The rich factor is the ratio of the number of DEGs to that of all genes annotated to a pathway term. A higher rich factor indicates greater intensity. [file Image_6.TIF]
